# Clostridium septicum infection of hepatic metastases following alcohol injection: a case report

**DOI:** 10.1186/1757-1626-2-9408

**Published:** 2009-12-31

**Authors:** Neam Saleh, Muhammad R Sohail, Rayhan H Hashmey, Mohammed Al Kaabi

**Affiliations:** 1 Department of Medicine, Division of General Medicine, Tawam Hospital, PO Box 15258, Al Ain, UAE; 2 Department of Medicine, Division of Infectious Disease, Tawam Hospital, PO Box 15258, Al Ain, UAE; 3 College of Medicine, Mayo Clinic, Rochester, MN, USA; 4 Department of Pathology, Division of Microbiology, Tawam Hospital, PO Box 15258, Al Ain, UAE

## Abstract

*Clostridium septicum *infections are generally associated with gastrointestinal or hematologic malignancies. We report the first case of hepatic metastases infection with *Clostridium septicum *that followed alcohol injection of liver lesion. Clinicians should consider this possibility in patients with underlying malignancy who present with hepatic abscess, as prompt surgical drainage and empiric antibiotics may be life saving.

## Background

Clostridia are gram positive, anaerobic, spore- forming bacilli that are ubiquitous in the environment and many are constituents of normal human gastrointestinal tract flora [1]. *Clostridium septicum *is one of the major species and is usually reported in association with spontaneous or non-traumatic gas gangrene [2]. Infection of hepatic metastases is a rare complication of *C. septicum *bacteremia [3].

Here, we report a case of *C. septicum *hepatic abscess at the site of liver metastases from adenocarcinoma of colon. To our knowledge, this is the first report of *C. septicum *infection following alcohol injection of hepatic metastases. We also reviewed the published English-language medical literature of hepatic metastases infection with *C. septicum*.

## Case presentation

A 53-year-old Lebanese man of Palestinian origin was admitted to an outside hospital with 5 days history of fever, cough and malaise. Patient's past medical history was relevant for ischemic cardiomyopathy and left sided adenocarcinoma of colon (Duke C), diagnosed 3 years prior to his current admission and treated with hemicolectomy and chemotherapy. Bilateral malignant pleural effusions and liver metastases were diagnosed during the follow up visits, for which he underwent percutaneous, intra-hepatic, injections of alcohol for ablation of liver lesions. Six weeks after the last alcohol injection, patient presented to emergency room with fever, cough, elevated hepatic transaminases and high C-reactive protein (CRP). Chest and abdominal computerized tomography (CT) showed multiple liver metastases and an 11 cm × 8.6 cm cavitating liver mass with air bubbles in the center (suggestive of abscess), bilateral pleural effusions with atelectasis and parenchymal lung lesions suggestive of pulmonary metastasis. The patient was started on an oral fluoroquinolone and referred to our hospital for further investigation and treatment.

**Figure 1:**
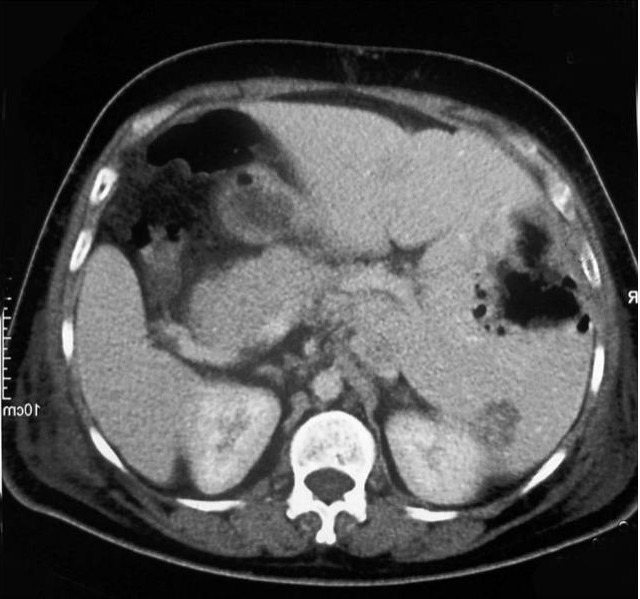
**Abdominal CT showing multiple liver masses, largest one being 11 cm × 8.6 cm with abscess formation**.

**Figure 2:**
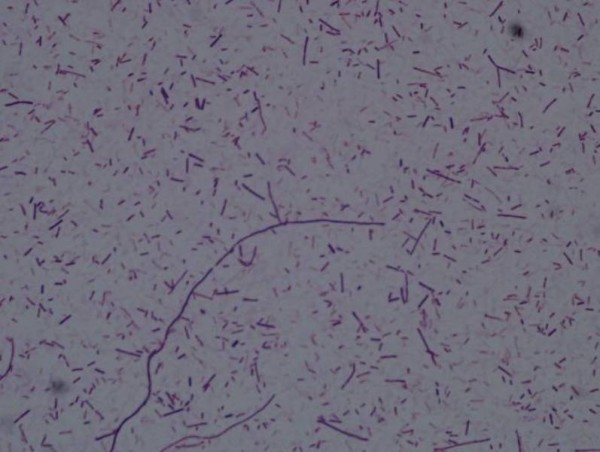
**Gram stain of hepatic abscess aspirate showing numerous gram-positive bacilli (oil immersion with 1000*x *objective)**.

Upon arrival to our hospital, patient was febrile (38°C), pale and ill looking. Blood pressure was (106/60 mmHg). Chest examination revealed bilateral rales and decrease air entry at the bases. No murmur was noted on cardiac examination. Abdomen was soft and non-tender, without hepatosplenomegaly. Patient had bilateral lower extremity pitting edema but no calf tenderness, joint swelling or skin rash was noted. Initial investigations revealed anemia (hemoglobin 10.6 g/dL), normal leukocyte (6.9 × 10^6^/L, 81% neutrophils) and platelets count (226 × 10^9^/L) and elevated (CRP of 267 mg/L). Electrolytes were normal. Liver function tests abnormal (alkaline phosphatase of 128 U/L, GGT 28 U/L, AST 244 U/L and ALT 141 U/L). Tumor marker screen showed elevated carcinoembyronic antigen (CEA) level of 139.3. A repeat CT of chest and abdomen at our hospital showed no significant changes compared with earlier imaging at the referring hospital (Figure 1). Patient was empirically started on piperacillin-tazobactam and vancomycin, which were later changed to the combination of ertapenem and caspofungin due to persistent fever. Repeat thoracentesis confirmed malignant cells on pleural fluid cytology. Stains and cultures were negative for bacteria and fungi. Blood and urine culture were negative as well. A CT-guided percutaneous drainage of liver abscess was performed that revealed malignant adenocarcinoma cells and the cultures grew *Clostridium septicum *(Figure 2) that was susceptible to penicillin, clindamycin, metronidazole, and carbapenems. Patient was switched to intravenous amoxicillin-clavulanate after culture results were available. Colonoscopy was performed two weeks later and showed multiple polyps and a large colonic mass confirmed as moderately differentiated adenocarcinoma on biopsy. Patient had gradual clinical improvement and was discharged home on oral amoxicillin-clavulanic acid after receiving the first cycle of Rituximab and 5-Fluorouracil. Patient was readmitted 3 weeks later and had palliative resection of the transverse colon for near-obstructing tumor recurrence at the anastomosis site. He was initially started on intravenous piperacillin-tazobactam, but then shifted to meropenem during to ongoing fever. However, blood and urine cultures were unrevealing at this admission and patient was discharged home on one week course of oral metronidazole and ciprofloxacin. Last follow up was 7 month later in the Oncology clinic to receive his ninth cycle of chemotherapy. Patient was afebrile, off antibiotics and had no evidence of recurrence of hepatic abscess on follow-up CT scan.

## Discussion

*Clostridium septicum *was the first pathogenic anaerobe cultured by Pasteur and Joubert. This organism reportedly caused much of the gas gangrene in wounded soldiers during the First and Second World Wars [4]. Disruption of colonic mucosa and reduction in neutrophil number or function are major risk factors for metastatic *C. septicum *infection [1]. Majority of *C. septicum *infections have been reported in patients with gastrointestinal tract malignancies (especially right sided adenocarcinoma of colon) and other disorders resulting in disruption of mucosal barriers such as severe enterocolitis or hemolytic uremic syndrome. Aortitis, gas gangrene, myonecrosis, septic arthritis and osteomyelitis, central nervous system infection, panopthalmitis and intra-abdominal abscesses are all reported complication of infections caused by *C. septicum*. Infection of hepatic metastases is a rare syndrome associated with *C. septicum*. We report the first case of *C. septicum *liver abscess following alcohol injection of liver metastasis and review the previously published cases of hepatic metastases infection with *C. septicum *(Table S1, Additional file 1).

The severity of clostridial infections is due to organism's motility and the ability to survive in anaerobic environment [1]. Also, *C. septicum *produces several toxins including lecithinase, deoxyribonuclease, hyaluronidase and hemolysin that disrupts cell membranes and induces occlusive microvascular thrombi resulting in tissue necrosis. Hepatic metastases provide ideal growth conditions for *C. septicum *due to poor blood supply and frequently a necrotic center with anaerobic milieu. Clostridia produce hydrogen and nitrogen gases at the site of infection which can be seen on plain radiographs or scans [1]. Clostridial infections carry high morbidity and mortality rate, up to 56% in one review by Larson et al [5]. Patients, who present with septic shock, have underlying liver cirrhosis or immunosuppression and those with delayed antibiotic administration have poor prognosis.

Most of the cases in our review (8/10, 80%) had hepatic metastases from gastrointestinal primary (Table S1, Additional file 1). Remaining two cases had primary cancer involving breast [6] and liver metastases from choriocarinoma [7]. Chemotherapy resulting in mucosal ulceration and neutropenia were the most common risk factor for *C. septicum *infection of liver metastases. In one case [8], infection was preceded by hepatic intra-arterial infusion of chemotherapy complicated by hepatic artery thrombosis, thus providing anaerobic conditions for clostridial growth.

Mean age of the patients was 51 years (range, 33 to 68 years). Fever, abdominal pain and elevated hepatic enzymes was the most common presentation. Four patients were in septic shock at initial presentation. Most (9/10, 90%) had positive blood cultures with *C. septicum*. In our patient, blood cultures were negative, as he had received antibiotics in outside hospital prior to obtaining cultures. However, cultures from abscess aspirate had heavy growth of *C. septicum*, highlighting poor penetration of antibiotics in these necrotic abscesses and need for drainage to achieve cure. Two patients [9,10] had multiple organism isolated from hepatic abscess in addition to *C. septicum*. Multiple hepatic abscesses were noted on abdominal computed tomography (CT) or ultrasound (US) in majority of patients. All except two patients [7,8] had drainage of liver abscesses (5 percutaneous and 3 surgical drainage). Of the two patients with conservative management, first patient with metastatic adenocarcinoma of colon [8] died six weeks after initial presentation while second with choriocarcinoma [7] was alive at two year follow-up visit.

Penicillin G is considered the drug of choice for most clostridial infections. Carbapenems, extended-spectrum cephalosporins and metronidazole are the usual alternatives. Most patients in our review were treated with a beta-lactam antibiotic, with or without clindamycin or metronidazole for additional anaerobic activity. Addition of a protein synthesis inhibitor, such as clindamycin, may help to reduce toxin production and resulting damage to hepatocytes [11]. In our review, there was no clear advantage of any particular antimicrobial regimen over the other, however numbers were too small to draw any meaningful conclusions. Four patients (40%) died during initial hospitalization for *C. septicum *hepatic abscesses. In patients where follow-up after discharge was reported, length of survival ranged from 37 days to 2 years.

## Conclusion

In conclusion, *C. septicum *infection should be suspected in patients who present with abscesses at the site of metastatic liver lesions. If unknown, a search for underlying malignancy ought to be performed. Systemic chemotherapy, hepatic artery thrombosis or alcohol injection, like in our case, may be additional risk factors for seeding of hepatic lesions by *C. septicum*. Early recognition and aggressive treatment, including percutaneous or open drainage and parenteral antibiotics directed against clostridia should be promptly initiated.

## Abbreviations

CEA: carcinoembryonic antigen; CRP: C reactive protein; CT: computerized tomography.

## Consent

Written informed consent was obtained from the patient for publication of this case report and accompanying images. A copy of the written consent is available for review by the Editor-in-Chief of this journal.

## Competing interests

The authors declare that they have no competing interests.

## Authors' contributions

NS extracted data from the patient's file, performed a literature search on the subject and was the major contributor in drafting the manuscript. RH and MS further refined the literature search, edited the case presentation and discussion, drafted the abstract and conclusions, formatted the paper and readied it for submission. ML provided pictures of the gram-stains of the hepatic abscess aspirate.

All authors have read and approved the final manuscript.

## Supplementary Material

Additional file 1 **Table S1**. Summary of cases with *Clostridium septicum *infection of Hepatic Metastases Click here for file

## References

[B1] Lorber B Mandell GL, Bennett JE, Dolin R Gas Gangrene and Other Clostridium-Associated Diseases Principles and Practice of Infectious Diseases 2005 6 Philadelphia, PA: Elsevier 2828 28

[B2] Stevens DL Musher DM Watson DA Eddy H Hamill RJ Gyorkey F Rosen H Mader J Spontaneous, nontraumatic gangrene due to Clostridium septicum Rev Infect Dis 1990 12 2 286 296 2330482 10.1093/clinids/12.2.286

[B3] Khan AA Davenport K A reminder of the association between Clostridium septicum and colonic adenocarcinoma Int Semin Surg Oncol 2006 3 12 10.1186/1477-7800-3-12 16646975 PMC1459865

[B4] MacLennan JD The histotoxic clostridial infections of man Bacteriol Rev 1962 26 177 276 14468017 PMC441149

[B5] Larson CM Bubrick MP Jacobs DM West MA Malignancy, mortality, and medicosurgical management of Clostridium septicum infection Surgery 1995 118 4 592 597 10.1016/S0039-6060(05)80023-6 7570310

[B6] Thel MC Ciaccia D Vredenburgh JJ Peters W Corey GR Clostridium septicum abscess in hepatic metastases: successful medical management Bone Marrow Transplant 1994 13 4 495 496 7517261

[B7] Lee CH Hsieh SY Case report: Clostridium septicum infection presenting as liver abscess in a case of choriocarcinoma with liver metastasis J Gastroenterol Hepatol 1999 14 12 1227 1229 10.1046/j.1440-1746.1999.02034.x 10634163

[B8] D'Orsi CJ Ensminger W Smith EH Lew M Gas-forming intrahepatic abscess: a possible complication of arterial infusion chemotherapy Gastrointest Radiol 1979 4 2 157 161 10.1007/BF01887516 456830

[B9] Kolbeinsson ME Holder WD Aziz S Jr Recognition, management, and prevention of Clostridium septicum abscess in immunosuppressed patients Arch Surg 1991 126 5 642 645 2021349 10.1001/archsurg.1991.01410290120024

[B10] Kahn SP Lindenauer SM Wojtalik RS Hildreth D Clostridia hepatic abscess. An unusual manifestation of metastatic colon carcinoma Arch Surg 1972 102 2 209 212 10.1001/archsurg.1972.04180020089018 5008917

[B11] Stevens DL Maier KA Mitten JE Effect of antibiotics on toxin production and viability of Clostridium perfringens Antimicrob Agents Chemother 1987 31 2 213 218 2882731 10.1128/aac.31.2.213 PMC174694

