# Supplementary material for: Clostridium septicum infection of hepatic metastases following alcohol injection: a case report
Source: Cases J. 2009 Dec 31;2:9408. doi: 10.1186/1757-1626-2-9408 (PMC2806403; doi:10.1186/1757-1626-2-9408)
Supplement: Additional file 1 — Table S1. Summary of cases with Clostridium septicum infection of Hepatic Metastases [file 1757-1626-2-9408-S1.DOC]

**Table 1.** Summary of cases with *Clostridium septicum* infection of Hepatic Metastases

| **Reference** | **Age (yr)/**  **Gender** | **Primary cancer/ location** | **Comorbid conditions** | **Clinical presentation** | **Drainage procedure** | **Antibiotics** | **Outcome [Follow-up]** |
| --- | --- | --- | --- | --- | --- | --- | --- |
| Saleh et al [current report] | 53/M | Metastatic adenocarcinoma of colon | Ischemic heart disease, cardiomyopathy, s/p hemicolectomy and chemotherapy. Liver metastases treated by alcohol injection | Fever, cough, hypotension, high CRP and LFTs. Multiple liver abscesses on CT scan. Positive abscess culture, Negative blood cultures | Percutaneous drainage of liver abscess | Piperacillin-tazobactam, followed by oral amoxicillin-clavulanic acid | Survived [7 months] |
| Kurtz et al [12] | 50/F | Metastatic colon cancer including liver metastases | s/p chemotherapy | Fever, painful, hepatomegaly and high CRP. Abdominal CT showed multiple liver abscesses. Positive blood and liver abscess cultures | Percutaneous drainage of liver abscess | Piperacillin-tazaobatam followed by imipenem | Died |
| Urban et al [13] | 68/M | Metastatic adenocarcinoma of colon with liver and lung metastases | S/P chemotherapy | Fever, nausea, abdominal pain, pleuritic chest pain, hypotension, leukocytosis, abdominal distension. Liver abscesses with air-fluid level and pneumoperitoneum on CT scan. Positive blood cultures | Percutaneous drainage of liver abscess | Broad spectrum intravenous antibiotics followed by oral penicillin suppression | Survived [37 days] |
| D’Orsi et al [8] | 52/F | Metastatic hepatic Adenocarcinoma of gastrointestinal primary | S/P hepatic arterial infusion of chemotherapy for liver metastases complicated by hepatic artery thrombosis | Fever, chills, hypotension, Liver abscess on plain radiographs. Positive blood cultures. | None | Cefalexin, clindamycin and gentamicin followed by oral chloramphenicol | Died [6 weeks after initial presentation] |
| Khan et al [3] | 59/M | Adenocarcinoma of colon with liver metastases | Hypertension | Fever, abdominal pain, anemia, elevated LFTs and renal failure. Liver abscesses confirmed by CT and US. Positive blood and abscess cultures. | Percutaneous drainage of liver abscess | Not specified | Survived [NA] |
| Lee et al [7] | 33/F | Choriocarcinoma with liver metastases | S/P dilatation and curettage for vaginal bleeding | Fever, chills, abdominal tenderness, leukocytosis, anemia and elevated Alk phos. Liver abscesses on CT scan and US. Positive blood cultures. | Drainage attempted | Cefamezine, gentamicin and metronidazole | Survived [2 years] |
| Thel et al[6] | 39/F | Metastatic breast cancer (stage IV) including liver metastases | S/P chemotherapy and BMT | Fever, tachycardia, tachypnea, abdominal pain, blood diarrhea and neutropenia. Abdominal CT showed two liver abscesses. Positive blood cultures. Negative abscess cultures | Percutaneous drainage of liver abscess | Ceftazidime followed by imipenem and clindamycin | Survived [2 months] |
| Kahn et al[10] | 44/M | Adenocarcinoma of colon with liver metastases | NA | Fever, abdominal pain, diarrhea, confusion, pleural effusion. Positive blood cultures. Polymicrobial infection of liver metastases. | Surgical drainage of abscess | Penicillin G, clindamycin, metronidazole | Died of aspiration pneumonia 2 months later |
| Kolbeinsson et al[9] | 57/M | Adenocarcinoma of colon with liver metastases | Perforates sigmoid cancer | Fever, abdominal pain and tenderness, leukocytosis, hypotension. Positive blood cultures. Polymicrobial liver abscess cultures. | Surgical drainage | Penicillin G, gentamicin, clindamycin | Died due to relapse of infection |
| Sarmiento et al[14] | 57/M | Adenocarcinoma of Colon with hepatic metastasis | Perforated transverse colon cancer | Febrile, septic, abdominal pain and tenderness. Positive blood cultures. | Surgical drainage | Not specified | Survived [NA] |

**Abbreviations**

Alk phos, alkaline phosphatase; BMT, bone marrow transplant; CRP, C - reactive protein; CT, computed tomography; F, female; LFTs, liver function tests; M, male; NA, not available; S/P, status post; US, ultrasound.
